# Proline-rich protein 11 overexpression is associated with a more aggressive phenotype and poor overall survival in ovarian cancer patients

**DOI:** 10.1186/s12957-020-02077-2

**Published:** 2020-12-04

**Authors:** Yu Zhan, Xueyuan Wu, Gang Zheng, Jingjing Jin, Chaofu Li, Guanzhen Yu, Wenfeng Li

**Affiliations:** 1 Department of Ultrasound, The First Affiliated Hospital of Wenzhou Medical University, Zhejiang, Wenzhou 325000 China; 2 Department of Chemoradiotherapy, The First Affiliated Hospital of Wenzhou Medical University, Zhejiang, Wenzhou 325000 China; 3 Department of orthopedics, The Second Affiliated Hospital of Wenzhou Medical University, Zhejiang, Wenzhou 325000 China; 4 Department of Oncology, Dalian Medical University, Dalian, Liaoning 116000 China; 5 Department of Oncology, Longhua Hospital Affiliated to Shanghai University of Traditional Chinese Medicine, Shanghai, 201203 China

**Keywords:** Ovarian cancer, Proline-rich protein 11, Cancer prognosis, Immunohistochemistry, N-cadherin, Early growth response protein 1

## Abstract

**Background:**

The proline-rich protein 11 (PRR11) is a newly identified oncogene associated with a poor prognosis in several human cancers. Nonetheless, research on its role in ovarian cancer (OC) remains largely understudied. Therefore, this study aims to evaluate the expression levels of PRR11 protein and its role in human ovarian cancer.

**Methods:**

Immunohistochemistry analysis was used to evaluate the expression levels of PRR11 protein in human samples obtained from 49 patients diagnosed with OC and subjected to curative surgery in the First Affiliated Hospital of Wenzhou Medical University between 2007 and 2015.

**Results:**

In total, 57.1% of the primary OC tumor tissue evaluated demonstrated overexpression of PRR11. Meanwhile, the survival analysis showed that the overall survival (OS) of patients presenting overexpression of PRR11 was significantly lower than the OS of the patients with negative PRR11. In subsequent experiments, it was found that silencing the expression of PRR11 expression inhibited the proliferation of tumor cells and the migration of cells in vitro. Further, cells subjected to PRR11 knockdown exhibited a decrease in tumor growth in vivo. The downregulation of PRR11 was coupled with a decrease in N-cadherin and downregulation in the expression of early growth response protein 1 (EGR1).

**Conclusions:**

The findings suggest that PRR11 might be considered as a potential target for prognostic assessment and gene therapy strategies for patients diagnosed with OC.

**Supplementary Information:**

The online version contains supplementary material available at 10.1186/s12957-020-02077-2.

## Introduction

Ovarian cancer (OC) is the most fatal gynecologic cancer and the sixth leading most aggressive cancer in women, accounting for up to 14,070 mortalities annually in the USA [1]. Due to the absence of specific symptoms, the majority of patients with OC often go undetected until the tumor has spread to the pelvic cavity or adjacent organs. Notably, 60% of patients are diagnosed at an advanced clinical stage [2]. Reports indicate that the clinicopathological features of OC including, advanced stage, incomplete surgery, residual volume after surgery, and tumor histological type are correlated with a poor prognosis [3–5]. Despite the new emerging therapeutic strategies targeting OC, recurrence remains the most critical threat to the long-term survival of patients with OC.

Recent studies have identified many prevalent genes in cancer patients responsible for poor prognosis and refractory or resistance to adjuvant therapy. They include TP53 [6], *PTEN* [7], *RB1* [6], *CCNE1* [6, 8, 9], and *HER2*. Besides, proline-rich protein 11 (PRR11) has been newly identified as a novel oncogene in lung cancer [10], tongue squamous cell carcinoma [11], and pancreatic cancer [12]. Additional studies have shown that PRR11 exhibits multiple biological functions, including cell cycle regulation and promotion of cellular migration and invasion [10]. Moreover, PRR11 has been regarded as a potential biomarker target for cancer treatment in the future, since it shows a significant association with poor prognosis in human cancers, such as gastric cancer [13] and cholangiocarcinoma [14]. Whilst acknowledging the evidence showing that PRR11 promotes the development of OC [15], the precise value of PRR11 in mediating the aggressive phenotype and poor overall survival (OS) of patients with OC is still unclear.

Herein, we conducted a retrospective study of patients diagnosed with OC and performed in vitro experiments geared towards clarifying the prognostic significance of the PRR11 protein in the development of OC.

## Materials and methods

### Patient and tissue specimens

A total of 49 patients with primary invasive ovarian neoplasms who underwent curative surgery in the First Affiliated Hospital of Wenzhou Medical University between 2007 and 2015 were enrolled in this study. The specific surgical procedure of each patient is shown in Supplementary Table 1 [16]. The peritoneal cancer index (PCI) was considered as the standard for describing carcinomatosis of colorectal cancer and mesothelioma. Regardless of tumor histologic origin, the PCI has been used to describe and explain the patients’ tumor spread pattern and disease severity. Based on previous literature [16], it was found the PCI score and lesion scope influence the prognosis of patients. The patients enrolled in this study were followed-up by postoperative imaging and hematological examination to track the progress of the disease and the prognosis of patients. To re-evaluate the pathological information, two expert pathologists were invited to assess PRR11staining intensity. According to the World Health Organization (WHO) classification criteria, these cases were classified as epithelial origin carcinomas. Including 32 cases of serous carcinoma (29 high-grade, 3 low-grade), 13 cases of endometrioid adenocarcinoma, 2 cases of mucinous carcinoma, and 2 cases of clear cell adenocarcinoma (Table 1). In addition, 5 cases of intraepithelial neoplasm and 3 cases of intravascular tumor emboli were enrolled. The median age of the patients was 56 years, ranging between 21 and 79 years. Based on the 2014 *Fédération Internationale de Gynécologie et d'Obstétrique* (FIGO) staging for OC, 10 cases were under stage I/II, while 39 cases were under stage III/IV. Stage I/II patients received a complete resection of the primary tumor, while the other patients, i.e., stage III/IV, underwent cytoreductive surgery, i.e., resecting the greater omentum and both ovaries with or without the uterus. After surgery, all patients received 6 to 8 cycles of chemotherapy (e.g., a taxane plus a platinum). For the 49 patients diagnosed with primary OC, clinical and follow-up data were collected for further survival analysis. The OS period was calculated from the day of initial surgery to the date the patient died or the date of the last follow-up visit. Also, 14 cases of benign epithelial lesions of the ovary were included as subject controls. Informed written consents were obtained from each patient. All methods and procedures were ethically approved by the Ethics Committee of the First Affiliated Hospital of Wenzhou Medical University in Zhejiang, China.

**Table 1:** Clinicopatological variables of ovarian cancer enrolled in this study

Total number = 49 (100%)	PRR11 positive	PRR11 negative
	28(57.1%)	21(42.9%)
Median age in years (range)	51.5 years (range: 21–74 years)	57 years (range: 35–79 years)
**Histology**		
Endometrioid	7(25.0%)	6(28.6%)
Mucinous	1(3.6%)	1(4.8%)
Serous	19(67.9%)	13(61.9%)
Clear cell adenocarcinoma	1(3.6%)	1(4.8%)
**FIGO stage**		
I/II	6(21.4%)	4(19.0%)
III/IV	22 (78.6%)	17(81.0%)
**Grade**		
Low grade	1(3.6%)	2(9.5%)
High grade	27(96.4%)	19(90.5%)
**PCI**		
median	15	15

### Tissue microarray construction

Paraffin-embedded tissue microarray (TMA) blocks were generated using a manual array (Beecher Instruments; Sun Prairie, Wisconsin, US). First, 1 mm of tissue cores were obtained in duplicate from each patient where the typical histological areas had been marked by pathologists. Then, consecutive sections, 4 μm in thickness, obtained from TMA blocks were prepared for immunohistochemical analysis.

### Immunohistochemistry and immunostaining analysis

After deparaffinization and rehydration routine procedures, TMA sections were processed for immunohistochemistry (IHC). Antibody antigen retrieval was performed using tissue submersion in citrate buffer (10 mM, pH 6.0) with heating. Sections were then incubated at 4 °C overnight with an anti-PRR11 antibody (HPA023923, Sigma-Aldrich; Saint Louis, MO, USA), anti-ki-67 (cat. 27309-1-AP, protein tech; Wuhan, China), anti-N-cadherin (cat. 610920, BD Biosciences; San Jose, CA, USA), and anti-early growth response protein 1 [EGR1] (ab54966, Abcam; Cambridge, UK). Subsequently, a two-step Envision kit (Agilent Technologies; Santa Clara, CA, USA) was used to visualize positive staining. Thereafter, the expression of PRR11, N-cadherin, and EGR1 proteins was evaluated by two researchers who were blinded to the study design using an Olympus CX31 microscope (Olympus Co., Tokyo, Japan). The antibodies were diluted with 1:100 goat serum for IHC analysis. Positivity was calculated by the semi-quantitative scoring system described by previous studies [14]. Generally, IHC staining intensity was assigned as: negative - 0; weak - 1; moderate - 2; and intense - 3. The percentage scores of IHC positive cells were set between 0 and 1 (0–100%). Theoretically, a weighted score ranging from 0 (0% of cells staining) to 3 (100% of the cells staining at 3+ intensity) was generated for each tissue core. An IHC score greater than (>) 0 was considered positive.

### PRR11 knockdown in vitro

The human OC cell lines including HO8910 and SKOV3, expressing the PRR11 protein, were purchased from the Chinese Academy of Sciences Cell Bank (Shanghai, China). Then, the expression of PRR11 was blocked through a stable transfection of a lentivirus expression plasmid that contained a small interference RNA targeting PRR11 (5′-ACGCAGGCCUUAAGGAGAATT-3′) [14]. The PRR11 knockdown plasmid was purchased from the GENECHEM Corporation (Shanghai, China). Also, an empty vector was transfected into cells, serving as the mock control group. Notably, knockdown efficiency of the targeted protein was evaluated by detecting the expression levels of PRR11 using Western blotting analysis.

### The Transwell migration assay

Both PRR11-knockdown cells and cells in the mock control group were harvested and introduced in the upper chamber of an 8-μm Transwell® (Corning; Corning, NY, US) containing serum-free Dulbecco’s modified Eagle’s medium (DMEM). Subsequently, 500 μl of DMEM media with 5% fetal bovine serum (FBS) was placed into the lower chamber. Then, the cells were allowed to migrate at 37 °C for 16 h after which the filters between the chambers were obtained for staining using 0.5% of the crystal violet stain. Afterward, the number of cells that migrated across the filters was manually counted under a phase-contrast microscope.

### Cell proliferation assays

Cells transfected with the PRR11-siRNA or with the empty vector were seeded in 96-well plates at a density of 5,000 cells per well. The CCK-8 kit (Dojindo Laboratories; Kumamoto, Japan) was then applied to assess the short-term proliferating rate of cells at 48 h in vitro. The sulforhodamine B (SRB) assay was subsequently applied to assess the long-term proliferating rate at 4–5 days in vitro.

### Western blot analysis

Whole-cell lysates were prepared using a cell lysis buffer containing a protease inhibitor cocktail (Sigma-Aldrich). The protein concentration was determined using a BioRad Protein Assay Kit (BioRad Laboratories; Hercules, CA, US) following the manufacturer’s protocol. Standard Western blotting analysis was performed using sodium dodecyl sulfate-polyacrylamide gel electrophoresis (SDS-PAGE) detected with anti-PRR11, anti-EGR1, and anti-N-cadherin antibodies using an enhanced chemiluminescence kit (Santa Cruz, CA, US). Loading of equal protein samples was monitored by probing the same membrane filter with an anti-β-actin antibody. Notably, antibodies were diluted at 1:1000 in BSA.

### Animal models

Cells subjected to PRR11-knockdown and control tumor cells were digested and suspended in phosphate-buffered saline [PBS] (1 × 107 cells in 0.1 mL of PBS). Then, cells were injected subcutaneously (sc) into the right flank of 4-week-old female Balb/c nude mice, respectively (each group, *n* = 5). Then, 2 weeks after the injection, all animals were sacrificed and the tumors were resected. The tumor volume (mm^3^) was calculated as *V* = 0.52 (length × width × depth). All animal experiments were approved by the Animal Ethics Committee of the Wenzhou Medical University.

### Statistical analysis

Statistical analyses were performed using the SPSS 16.0 software (IBM; Armonk, NY, US). The chi-squared (*χ*2) test was used for analyzing the association of categorical data. The Kaplan-Meier method was applied to estimate the OS while the log-rank test was used to assess survival differences between the groups. The dataset of the gene expression omnibus was GDS3592. A two-sided *P* value of less than 0.05 *P* < 0.05 was considered statistically significant.

## Results

### PRR11 expression in OC tissues

**Fig. 1:**
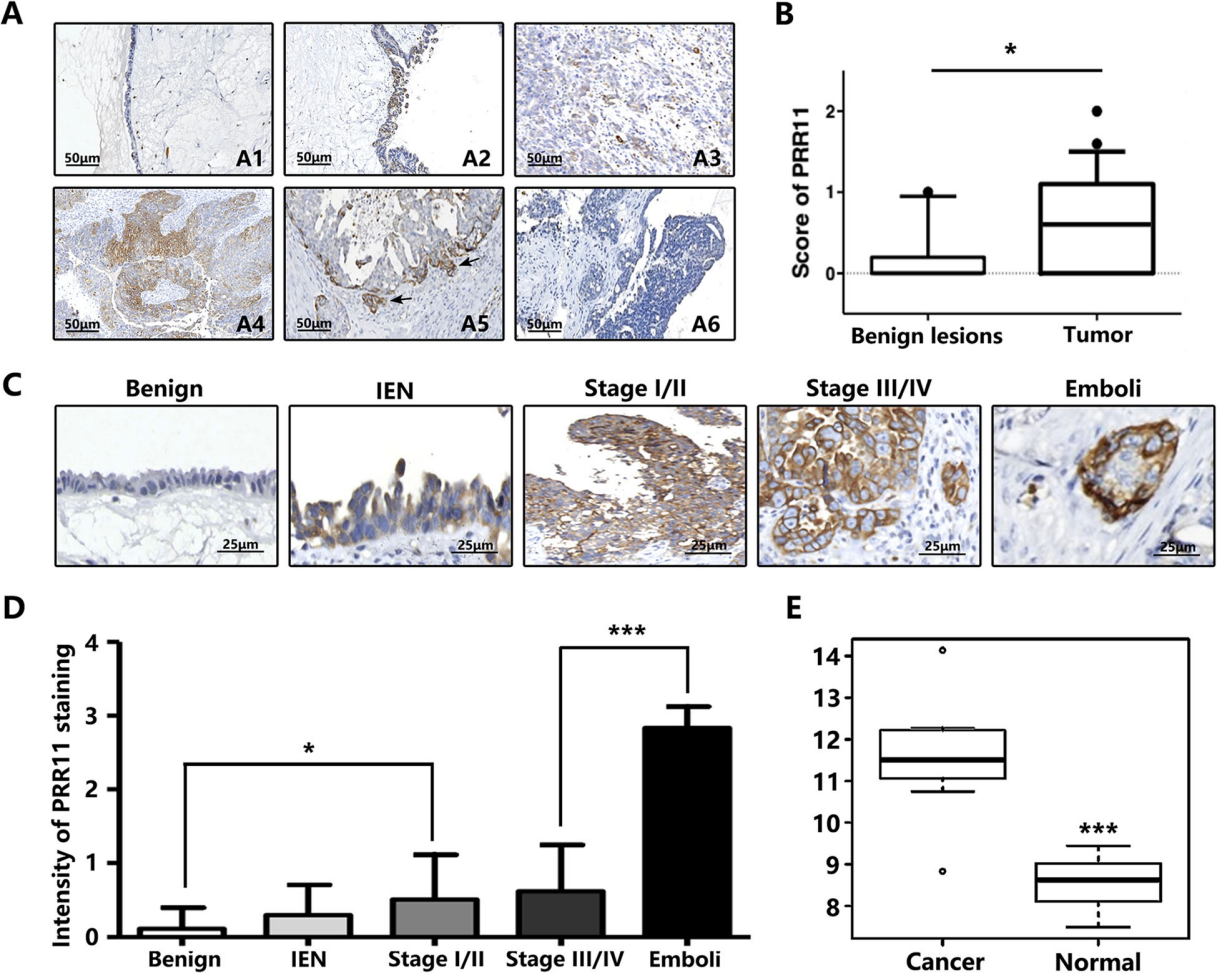
PRR11 expression in human ovarian cancer (OC) and non-cancerous tissues. **A-1** Negative staining of PRR11 in the epithelium of ovarian benign lesions. **A-2**, **A-3** Low expression of PRR11 in poorly-differentiated cancer cells. **A-4** High expression of PRR11 in endometrioid adenocarcinoma. **A-5** Strong staining of PRR11 in the front edge of serous carcinoma. **A-6** Negative staining of PRR11 in serous carcinoma. The original magnification is 200×. **b** Graphical representation of the intensity of PRR11 staining in cancer tumors and noncancerous tissues. **c** Expression of PRR11 in benign lesions, intraepithelial neoplasm (IEN), tumor at stages I/II, tumor at stages III/IV, and intravascular tumor emboli. **d** The intensity of the PRR11 staining increases with the progression of OC: 0.115 ± 0.29 in benign lesions, 0.32 ± 0.39 in intraepithelial neoplasm (IEN), 0.51 ± 0.60 in tumors at stages I/II, 0.62 ± 0.63 in tumors at stage III/IV, and 3-0.29 in intravascular tumor emboli. *P < 0.05, ***P < 0.001. **e** Gene expression omnibus (GEO) repository dataset (the data from GDS3592 data set) profiles revealed higher expression of PRR11 in OC epithelial cells than the expression observed in ovarian normal surface epithelia. *P < 0.05, ***P < 0.001

After immunostaining PRR11 in the OC tissues of 49 patients, a pale yellow or brown-yellow positive staining was observed in the clinicopathological data. Also, it was noted that the expression of PRR11 protein primarily occurred in the cell cytoplasm. Out of the 49 tumor samples, 28 (57.1% of the patients) showed a positive expression of PRR11 (Table 1). As shown in Fig. 1A, ovarian benign lesions showed limited staining (Fig. 1A–1), while PRR11 immunostaining was observed across the different pathological types of OC cells (Fig. 1A2–5), with varying staining intensity among the tissue status (Fig. 1A5–6). A comparison of the 49 tumor samples revealed only 3 out of 14 ovarian benign lesions cases with PRR11 positivity (21.4% of patients) (Fig. 1b). To evaluate the effects of PRR11 upregulation in OC tissues, the staining intensity of PRR11 in clinical stages I–IV of OC primary tumors was further analyzed (Fig. 1c). The intensity of the PRR11 staining had significant differences between benign lesions and stage I/II and between stage III/IV and intravascular tumor emboli of IHC as follows: 0.115 ± 0.29 in benign lesions, 0.32 ± 0.39 in intraepithelial neoplasm (IEN) lesions, 0.51 ± 0.60 in tumors at stage I/II, 0.62 ± 0.63 in tumors at stages III/IV, and 3–0.29 in intravascular tumor emboli (Fig. 1c-d). In addition, the datasets of gene expression omnibus (GEO) showed that the expression of PRR11 was significantly higher in OC epithelial cells compared to normal human tissues (Fig. 1e).

### Association of PRR11 expression with the overall survival of OC patients

**Fig. 2:**
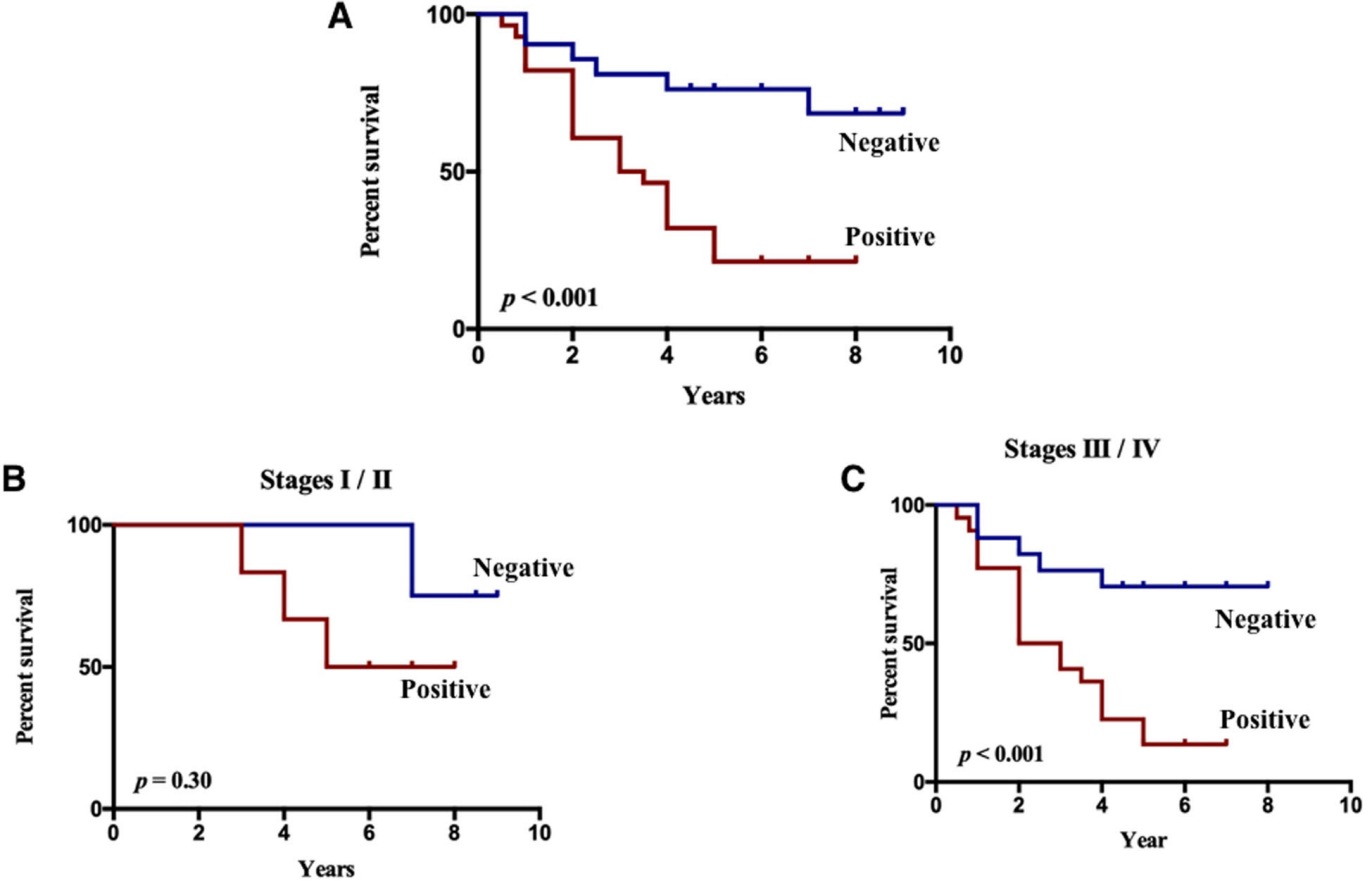
Kaplan-Meier curves of overall survival in patients with OC according to the expression of PRR11. **a** Patients with positive expression of PRR11 presented shorter overall survival (OS) duration than those patients negative for PRR11 protein expression (6.0 years versus 3.25 years, *P* < 0.001). **b** Subgroup analysis according to the FIGO tumor staging criteria showing that PRR11-positive patients at stages I/II present an unfavorable prognosis (median OS: 5.5 years) compared with PRR11-negative patients at stages I/II (median OS: 7.75 years, *P* = 0.30). **c** PRR11-positive patients at stages III/IV showed an extremely unfavorable prognosis (median OS: 2.5 years) compared with PRR11-negative patients at stage III/IV (median OS: 5.0 years, *P* < 0.001)

To determine the prognostic significance of the protein expression of PRR11, 49 OC patients with a median age of 56 years ranging between 21 and 79 years were divided into two cohorts based on whether PRR11 is expressed—the positive group and the negative group. The negative group comprised 21 OC patients while the positive group consisted of 28 OC patients. Notably, the median cumulative survival duration in these patients was 4.5 years (Supplementary Table 1). Survival analysis showed that patients who showed positive expression of PRR11 had significantly shorter OS duration than those with negative expression of PRR11 protein (6.0 years versus 3.25 years, *P* < 0.001; Fig. 2a). Besides, a subgroup analysis based on the FIGO stage of malignant tumors was conducted. It was found that PRR11-positive patients at stage III/IV showed an extremely unfavorable prognosis (median OS: 2.5 years), compared to PRR11-negative patients at stage III/IV (median OS: 5.0 years, *P* < 0.001; Fig. 2c). PRR11-positive patients at stage I/II showed an unfavorable prognosis (median OS: 5.5 years), compared to PRR11-negative patients at stage I/II (median OS: 7.75 years, *P* = 0.30; Fig. 2b). Surprisingly, no significant clinical factors which predict the prognosis of OC were identified using univariate analyses, which might be attributed to the limitation in the number of patients recruited in this study.

### PRR11 silencing inhibits cell proliferation and migration of OC cells in vitro and in vivo

**Fig. 3:**
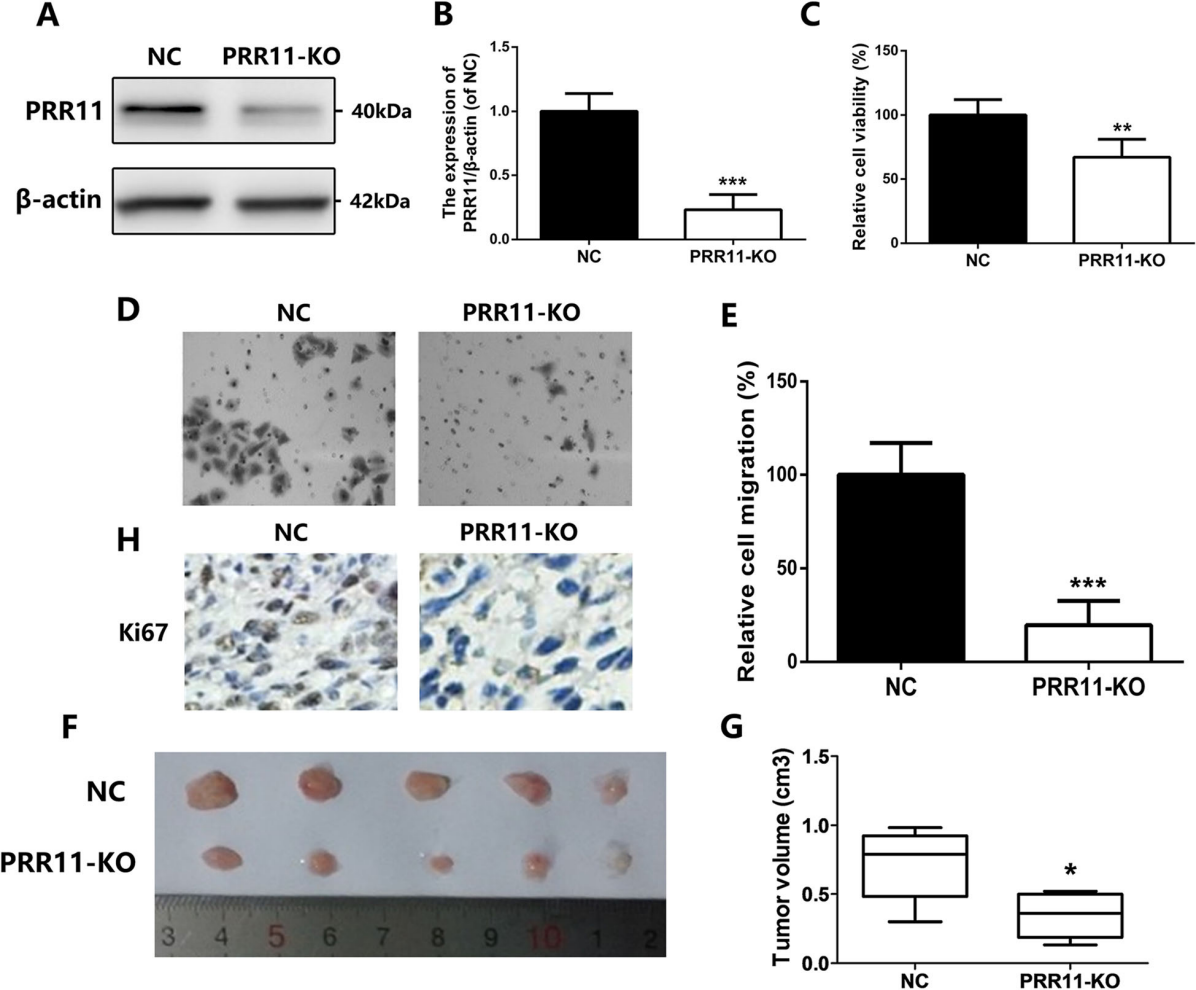
Knockdown of PRR11 expression inhibited the proliferation and migration of OC cells in vitro and in vivo. a Western blot and **b** qPCR analysis showing expression levels of the PRR11 protein in HO8910 and SKOV3 human OC cells after stable infection with shPRR11 viruses. **c** Relative cell viability of cancer cells after PRR11-knockdown. **d** Representative pictures of the cell migration assay and **e** graphical presentation of the number of migrating cells 16-h post-transfection. **f** Pictures of tumors resected from subcutaneous tumor xenograft models and **g** tumor volume. **h** Ki67 staining in tumor sections. *P < 0.05, **P < 0.01, compared with the control group

To confirm the biological effect of PRR11 expression in OC, PRR11-targeted siRNA was stably transfected into human OC cell-lines, including HO8910 and SKOV3. Then, western blot and quantitative polymerase chain reaction (qPCR) analysis was applied to test the efficiency of PRR1 knockdown (Fig. 3a-b). Results showed that knocking down the expression of PRR11 significantly blocked the growth of cancer cells (HO8910 and SKOV3) both in short-term proliferation (Fig. 3c) and long-term proliferation assays (Supplementary Fig. 2). Moreover, the migration capability of the cancer cells determined was significantly reduced after knocking down the expression of PRR11 (Fig. 3d-e). Furthermore, in vivo assay showed that silencing of PRR11 expression markedly inhibited the growth and volume of tumors as revealed by anatomical assays, as well as by the expression of the cellular marker for proliferation, Ki67 (Fig. 3f-h).

### Knockdown of PRR11 inhibits EGR1 and N-cadherin expression

**Fig. 4:**
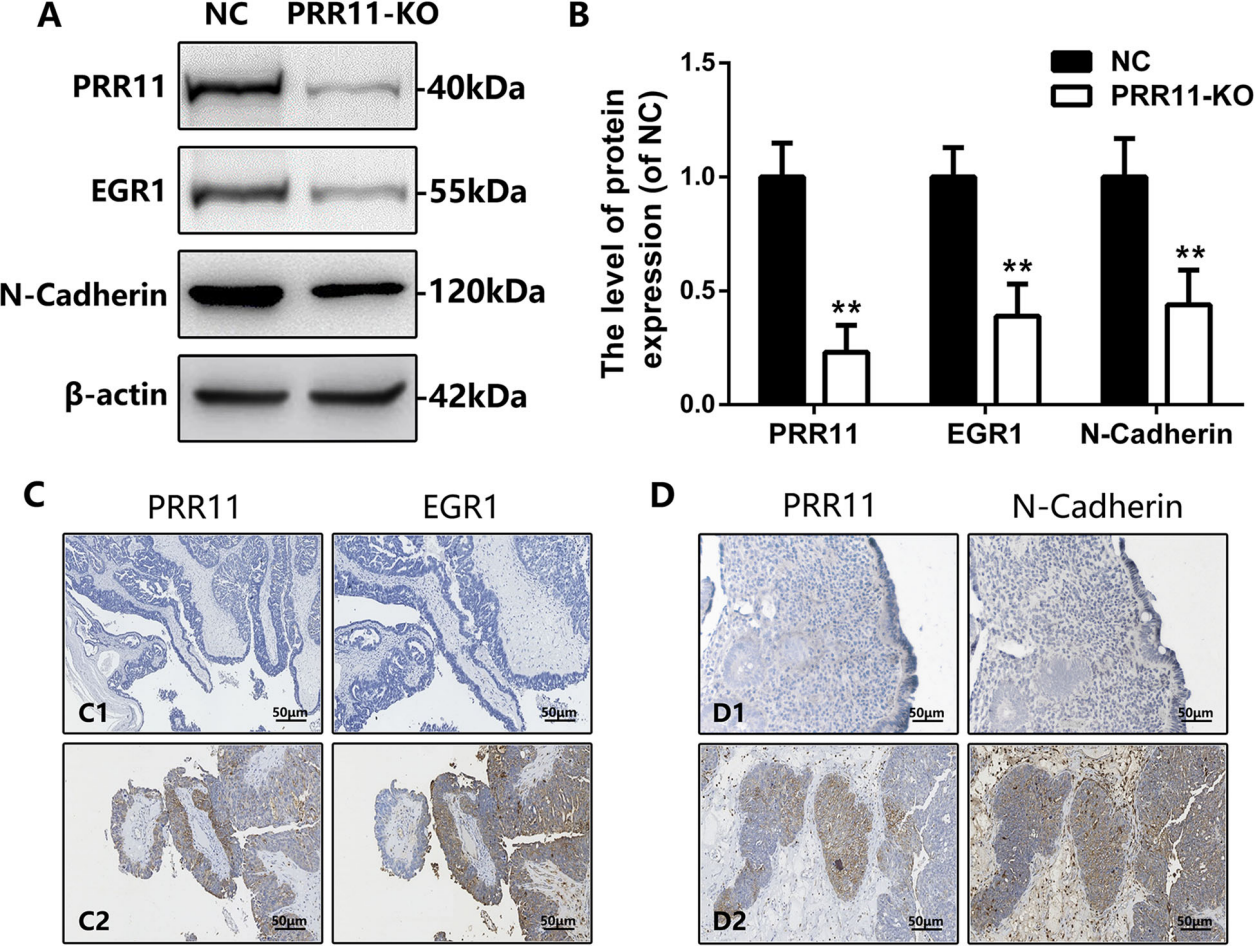
Correlation analysis among the expression levels of PRR11, N-cadherin, and EGR1. **a** Western blot analysis detecting the protein levels of N-cadherin and EGR1 after PRR11 was knockdown for 48 h. **b** Graphical representation showing a significant reduction of PRR11, N-cadherin, and EGR1 protein levels according to ß-actin baseline levels. **c** Co-localization of PRR11 and EGR1 in the same tissue samples. **c-1** Co-negative expression of PRR11 and EGR1 in the same tissue sample. **c-2** Co-positive expression of PRR11 and EGR1 in the same tissue sample. Original magnification is ×200. **d** Co-localization of PRR11 and N-cadherin in the same tissue sample. **d-1** Co-negative expression of PRR11 and N-cadherin in the same tissue sample. **d-2** Co-positive expression of PRR11 and N-cadherin in the same tissue sample. Original magnification is ×200.

EGR1 regulates cell survival, proliferation, and cell death [17]. A recent study reported that PRR11 signals could significantly induce the expression of EGR1 gene in patients diagnosed with hilar cholangiocarcinoma patients [14]. Therefore, this work detected the expression of EGR1 in established PRR11 knockdown cells. Additionally, silencing the expression of PRR11 protein caused a downregulation in the expression of EGR1 protein in vitro (Fig. 4a-b). In the cancer tissues analyzed, the staining observed for expression of both EGR1 and PRR11 protein nearly overlapped in the same regions, indicating a consistent expression pattern between these two markers (Fig. 4c-d). The correlation of the staining intensity between the expression of EGR1 and PRR11 was examined in different pathological tissues (the correlation coefficient *R* = 0.324), and it was found to be statistically different (*P* = 0.017).

Besides being one of the markers used to examine cell migration and invasion, N-cadherin is associated with epithelial-to-mesenchymal transition (EMT) cell processes [18]. Based on these findings, this study examined whether the knockdown of PRR11 might influence the EMT pathway. Interestingly, it was demonstrated that the downregulation of PRR11 induced a downregulation in the expression of N-cadherin (Fig. 4a-b). Further, it was observed that the expression of N-cadherin and PRR11 occurred in similar regions (Fig. 4e). However, the correlation of the staining intensity between these two proteins was statistically different in the OC pathological tissues (*R* = 0.424, *P* = 0.011).

### PRR11 expression may not be associated with PCI score in ovarian cancer patients

PCI is used to assess the extent of peritoneal cancer throughout the peritoneal cavity (FIGO IIIC/IV). Previous studies reported PCI scores can be used to evaluate in the prognosis of ovarian cancer patients [16]. Therefore, here, the correlation between survival time of ovarian cancer patients and PCI scores was statistically compared. As a result, patients with ovarian cancer and a higher PCI score exhibited a worse prognosis and a shorter survival time, whereas patients with ovarian cancer and a lower PCI score had a better prognosis (Supplementary Fig. 3A). Also, a comparison between the expression of PRR11 in patients with FIGO stages III and IV, and whether the expression of PRR11 is related to PCI score in ovarian cancer patients, was conducted, whether PRR11 affects the prognosis of ovarian cancer patients by regulating the PCI score. No significant difference was found in the PCI scores between patients with negative or positive PRR11 expression (Supplementary Fig. 3B). There also has no difference of OS between stage III/IV patients with PRR11 negative expression and stage I/II patients with PRR11-positive expression (Supplementary Fig. 3C). Therefore, this paper concludes that PRR11 has no direct relationship with PCI scores on the prognosis of ovarian cancer patients.

## Discussion

Cell cycle is a fundamental physiological process under the surveillance of numerous regulators and checkpoints to precisely keep cell division under control [19]. Importantly, uncontrolled cell cycle processes trigger the formation of tumors and subsequent development of cancer [19]. The PRR11 protein was first identified by Ji and colleagues in lung cancer as a protein that functions periodically and specifically promotes S-phase progression of the cell cycle [10]. Further high-throughput screening under PRR11 expression knockdown identified several cell cycle-related genes including cyclin A1 (CCNA1), the ribonucleotide reductase catalytic subunit M1 (RRM1), and the mitogen-activated protein kinase 4 (MAP4K4). These genes have been reported to promote the G1/S-phase progression of the cell cycle or the regulation of cyclin-dependent kinases (CDKs) [10, 20, 21]. Moreover, Zhang et al. clarified that PRR11 regulates the G2/M transition of the cell cycle and promote premature chromatin condensation (PCC) processes in cancer cells [22]. Recent studies have shown that PRR11 is implicated in the formation of gastric cancer [13], breast cancer [23], and hilar cholangiocarcinoma [14].

OC is the most dismal malignancy of the female gynecological system, with a prognosis that is much worse than breast cancer [5]. Despite the emergence of newly developed therapies, significant advancement in the prognosis of late-stage OC has not been reported [1]. Notably, the critical factors that influence prognosis are cell invasion and lymph node metastasis. Furthermore, numerous genes and pathways are responsive in ovarian carcinogenesis or can be regarded as clinical predictors for disease recurrence and patient survival [24, 25]. Zhu et al. previously reported that PRR11 participated in the progression and metastasis of OC through the PI3K/AKT/β-catenin signaling pathway [15]. Nonetheless, the molecular mechanisms underlying the poor OS rates observed in OC patients with high expression of PRR11 remain unclear.

Herein, we demonstrate that PRR11 is highly expressed in primary OC tumors. Moreover, the staining intensity of the PRR11 protein gradually increased along with the progression of OC, from low expression in benign tumors to intensive positivity in tumor emboli indicating that PRR11 might be used as an OC tumor biomarker. Previous studies have shown that PRR11 immunostaining can be found mainly in the cytoplasm and the plasma membrane of OC cells [15]. In several of our pathological images, PRR11 IHC analysis showed a strong staining on the cell membrane and in the cytoplasm, which was relatively weak perhaps due to marginal effects and differences between tissues. Based on survival analysis data, PRR11 might be a risk indicator of OC progression during the advanced stages of the disease. We showed that the expression of PRR11 had no statistical significance during the early stages of the ovarian cancer on survival analysis data though, which may because the number of subjects is too small to yield reliable results. Furthermore, our in vitro experiments showed that the knockdown of PRR11 causes cell growth arrest and migration reduction. Despite the increased need to unravel the molecular mechanisms and pathological features of PRR11 in the development of OC, existing studies have directed their focus to the expression of PRR11 in the pathological and clinical features of other cancer tumors. And based on our report, the association between PRR11 and PCI score in ovarian cancer may require further sample size expansion for in-depth analysis.

In the recent past, studies have shown that expression of PRR11 can be combined with its neighboring gene expression to predict the risk of their biochemical recurrence [26] or influence the S-phase of the cell cycle [10]. Nevertheless, the roles of PRR11 in the cellular EMT process in OC remain unclear. Our findings showed that knocking-down PRR11 in OC cells induced the downregulation of N-cadherin and EGR1. Notably, N-cadherin is a key indicator in cell migration and EMT processes; thus, a downregulation in expression of N-cadherin is accompanied by EMT. EGR1 is one of the immediate-early response genes with binding sites in promoter regions of many growth factor genes and proto-oncogenes [17]. We further revealed that EGR1 and N-cadherin, which were downregulated with the knockdown of PRR11 expression in HC, were significantly downregulated by the PRR11 knockdown in OC cells. In addition, Zhao and colleagues proposed that the downregulation of EGR1 in gastric cancer cells might influence EMT [27]. Therefore, we hypothesized that the PRR11 protein might promote EMT processes by regulating the levels of EGR1 and N-cadherin and might potentially promote the initiation of EMT [18].

## Conclusions

In conclusion, we demonstrate that PRR11 is highly upregulated in primary OC cells and is adversely associated with the clinical prognosis of OC in vitro, and the knockdown PRR11 caused cell growth arrest and inhibited cell migration. Additionally, knockdown of PRR11 caused a downregulation in expression levels of N-cadherin and EGR1 proteins. The above observations strongly suggest that PRR11 might be a promising indicator of predicting patient survival and drug target for the treatment of ovarian carcinoma.

## Supplementary Information

**Additional file 1:** **Supplementary Fig. 1.** PRR11 protein expression levels between serous carcinoma (1 ± 0.65) and endometrioid carcinoma (0.65 ± 0.61). NS: not significant.

**Additional file 2:** **Supplementary Fig. 2.** Long-term growth assay of HO8910 and SKOV3 OC cells. Cells were stably infected with shPRR11and shNT viruses and the cell viability was determined using the sulforhodamine B (SRB) cell proliferation and cytotoxicity assay kit using a 560 nm microplate reader. Error bar shows the data ± standard error (SE).

**Additional file 3:** **Supplementary Fig. 3.** PRR11 expression was not associated with PCI score in ovarian cancer patients. (A) the comparison of total survival time of ovarian cancer patients between PCI scores greater than 20 and less than 20; (B) The relationship of PCI scores between the PRR11 negative expression and PRR11 positive expression in patients with FIGO stage III and IV of ovarian cancer patients. C) The comparison of total survival time between the stage III/IV patients with PRR11 negative expression and the stage I/II patients with PRR11 positive expression.

## Data Availability

The datasets used and/or analyzed during the current study are available from the corresponding author on reasonable request.
