# Supplementary figures and images for: Proline-rich protein 11 overexpression is associated with a more aggressive phenotype and poor overall survival in ovarian cancer patients
Source: World J Surg Oncol. 2020 Dec 4;18:318. doi: 10.1186/s12957-020-02077-2 (PMC7718657; doi:10.1186/s12957-020-02077-2)

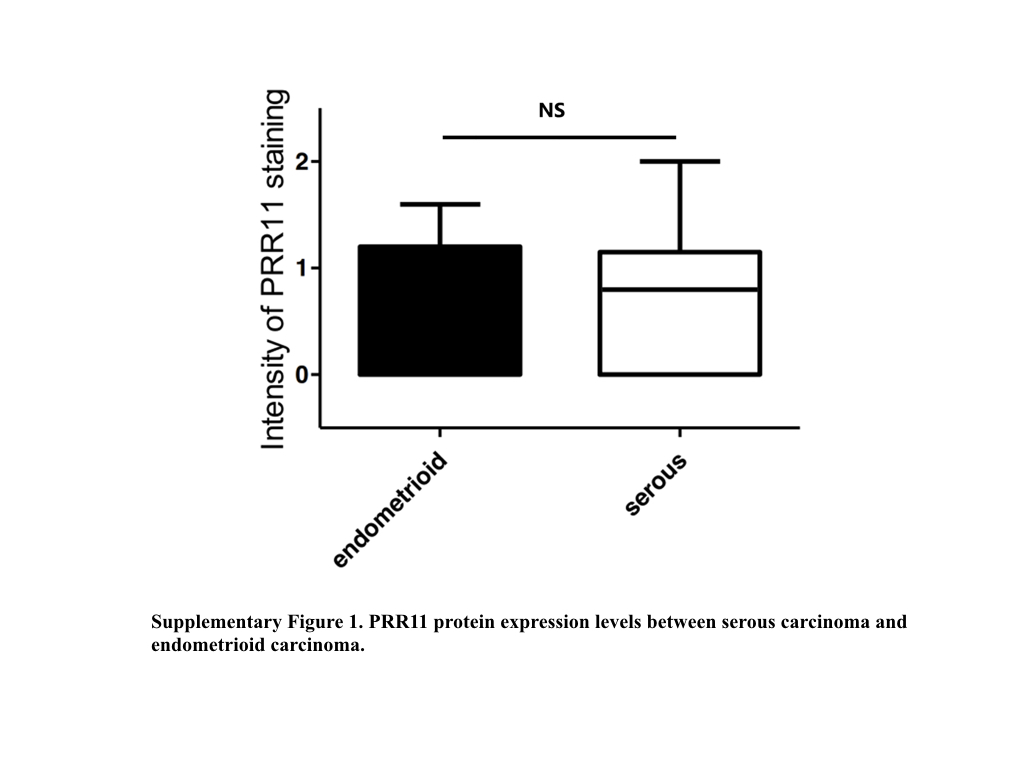

Supplement: Supplementary file 1 — Additional file 1: Supplementary Fig. 1. PRR11 protein expression levels between serous carcinoma (1 ± 0.65) and endometrioid carcinoma (0.65 ± 0.61). NS: not significant. [file 12957_2020_2077_MOESM1_ESM.jpeg]

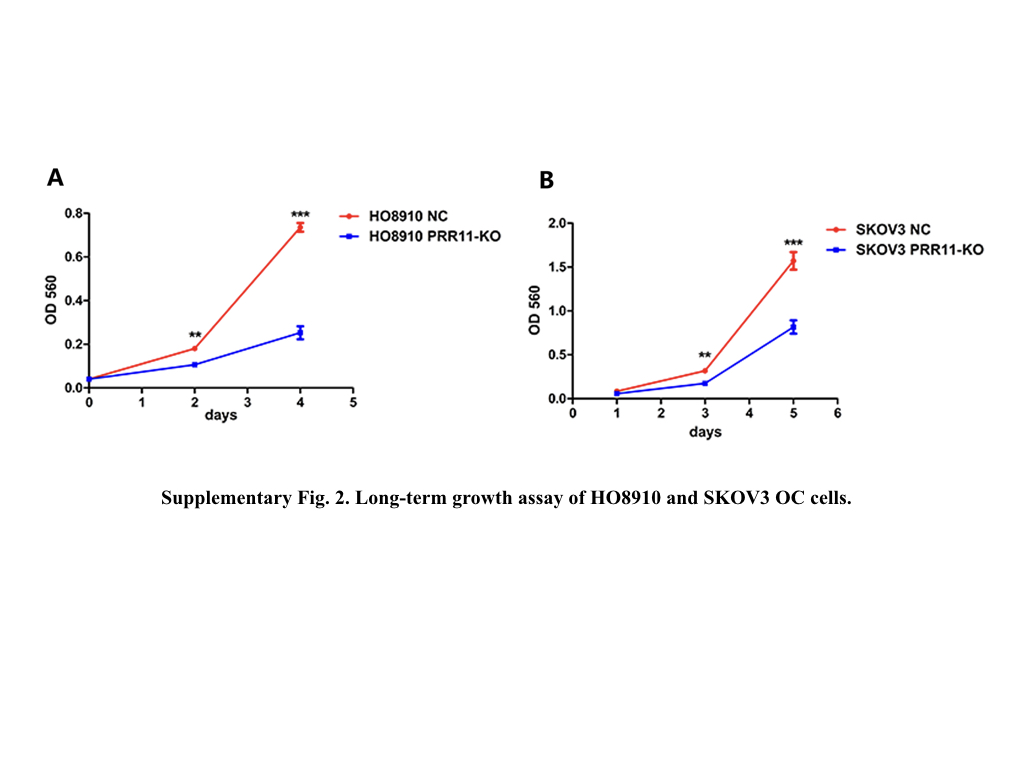

Supplement: Supplementary file 2 — Additional file 2: Supplementary Fig. 2. Long-term growth assay of HO8910 and SKOV3 OC cells. Cells were stably infected with shPRR11and shNT viruses and the cell viability was determined using the sulforhodamine B (SRB) cell proliferation and cytotoxicity assay kit using a 560 nm microplate reader. Error bar shows the data ± standard error (SE). [file 12957_2020_2077_MOESM2_ESM.jpeg]

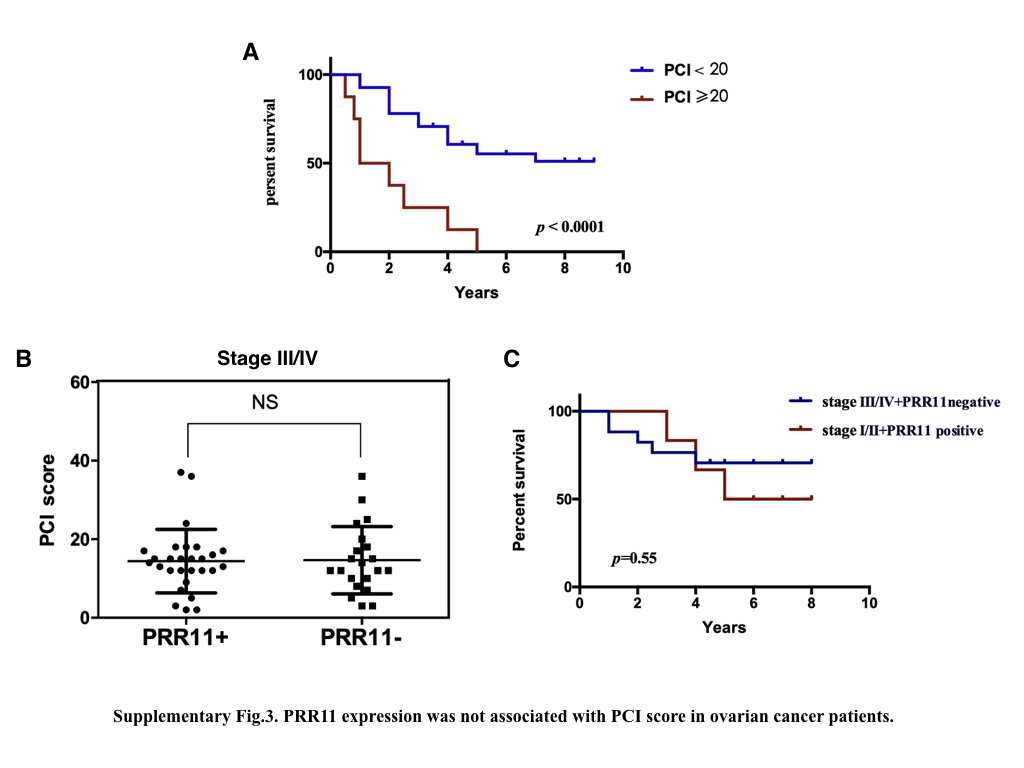

Supplement: Supplementary file 3 — Additional file 3: Supplementary Fig. 3. PRR11 expression was not associated with PCI score in ovarian cancer patients. (A) the comparison of total survival time of ovarian cancer patients between PCI scores greater than 20 and less than 20; (B) The relationship of PCI scores between the PRR11 negative expression and PRR11 positive expression in patients with FIGO stage III and IV of ovarian cancer patients. C) The comparison of total survival time between the stage III/IV patients with PRR11 negative expression and the stage I/II patients with PRR11 positive expression. [file 12957_2020_2077_MOESM3_ESM.jpeg]
